# One-year efficacy of Dupilumab in sense of smell, nasal polyp score and quality of life in CRSwNP patients: A real-world multicenter study in Brazil^☆^

**DOI:** 10.1016/j.bjorl.2025.101704

**Published:** 2025-09-06

**Authors:** Otávio Marana Mieli, Fabiana Cardoso Pereira Valera, Vanessa Ramos Pires Dinarte, Clara Mônica Figueiredo de Lima, Marcio Nakanishi, Maria Eduarda Trocoli Zanetti, Felipe Oliveira Pires, Denny Marcos Garcia, Adriana de Andrade Batista Murashima, Luisa Karla de Paula Arruda, Fabrizio Ricci Romano, José Eduardo Seneda Lemos, Eduardo Macoto Kosugi, Eulalia Sakano, Miguel Soares Tepedino, Edwin Tamashiro, Wilma Terezinha Anselmo-Lima

**Affiliations:** a Faculdade de Medicina de Ribeirão Preto – Universidade de São Paulo, São Paulo, SP, Brazil; b Faculdade de Medicina de Marília (FAMEMA), Marilia, SP, Brazil; c Hospital Universitário Professor Edgar Santos, Salvador, BA, Brazil; d Faculdade de Medicina da Universidade de Brasília, Brasilia, DF, Brazil; e Faculdade de Medicina da Universidade de São Paulo (FMUSP), São Paulo, SP, Brazil; f Escola Paulista de Medicina - Universidade Federal de São Paulo (EPM-UNIFESP), São Paulo, SP, Brazil; g Faculdade de Ciências Médicas da Universidade Estadual de Campinas (UNICAMP), Campinas, SP, Brazil; h Faculdade de Ciências Médicas, Universidade do Estado do Rio de Janeiro, Rio de Janeiro, RJ, Brazil

**Keywords:** AERD, Biologics, Chronic sinusitis, Loss of smell, Nasal polyps

## Abstract

**Introduction:**

Chronic rhinosinusitis with nasal polyp (CRSwNP) is a predominant type 2 inflammatory disease, affecting the sense of smell and quality of life. Loss of smell compromises physical and emotional health, creating negative impacts and its treatment in CRSwNP is challenging.

**Aim:**

To present the outcomes of dupilumab in olfactory function, Nasal Polyp Score (NPS) and quality of life in Brazilian patients with severe CRSwNP.

**Material & methods:**

We selected severe type 2 CRSwNP patients from different Brazilian centers. Dupilumab was indicated after failure of optimized medical and surgical treatment. Patients were evaluated with endoscopic Nasal Polyps Score (NPS), Connecticut smell test (CCCRC) and quality of life questionnaire (SNOT-22), at baseline (T0) and at one year (T1) after dupilumab was initiated. The clinical response was classified as good or non-responsive, according to the degree of improvement at T1. Regarding disease control, patients were classified as controlled, partially controlled or uncontrolled.

**Results:**

53 patients completed one year of treatment (19–76 years-old); 51 individuals (96.2%) had asthma and 33 had Aspirin-Exacerbated Respiratory Disease (AERD) (62.3%). Dupilumab decreased mean SNOT-22 scores from 61.9 to 16.7 points (paired *t*-test, *t* = 13.4, p < 0.0001). The median CCCRC scores improved from anosmia (0-points) to mild hyposmia (5.5-points) (Wilcoxon signed rank test, p < 0.0001). Regarding NPS, we observed a decrease from a median of 6 in T0 to 1 at T1. AERD patients showed similar responses to those with only asthma as comorbid disease. Overall, most of the patients (86.8%) showed improvement in the disease control (Bowker's Test, p < 0.0001).

**Conclusions:**

Real-world data show that dupilumab improved NPS, smell function, and SNOT-22 scores in the Brazilian CRSwNP population. These results support the benefits of dupilumab in treating severe cases of CRSwNP.

**Level of evidence:**

Level 3.

## Highlights

• Chronic Rhinosinusitis with Nasal Polyps (CRSwNP) impacting quality of life.
• Patients with CRSwNP presents nasal obstruction and loss of sense of smell.
• Real-world data show that dupilumab improved parameters in CRSwNP.
• Nasal polyp score, smell function, and quality of life test showed improvement.
• Results support the benefits of dupilumab in treating cases of CRSwNP in Brazil.

## Introduction

Chronic Rhinosinusitis with Nasal Polyps (CRSwNP), a predominantly type-2 inflammatory disease, is a common condition characterized by sinonasal inflammation lasting more than twelve weeks, with an estimated global prevalence of approximately 1%–5% of the general population.^1^, ^2^, ^3^, ^4^, ^5^

This disease significantly impacts patients’ quality of life, with progressive nasal obstruction being the most frequently reported complaint. Other symptoms include postnasal drip, anosmia or hyposmia. Moreover, CRSwNP patients frequently use multiple courses of systemic corticosteroids and antibiotics, often providing temporary symptom relief. In the more severe cases, despite multiple surgical procedures, symptoms usually persist.^6^

Evidence in the literature indicates that loss of smell impairs both physical and emotional health, contributing to social isolation and negatively affecting daily activities, interpersonal relationships, and functional performance. Additionally, smell loss is frequently associated with psychological disorders such as anxiety and depression.^6^ Sensory perception of the world around is lost.^6^^,^^7^ The underlying mechanisms of olfactory loss in CRSwNP are multifactorial. Smell loss may result from either conductive dysfunction (nasal obstruction) or neuroimmune dysfunction (damage of the olfactory system).^6^^,^^7^

Olfactory impairment is challenging to treat and may be the first sign of disease recurrence.^2^^,^^8^^,^^9^ The use of oral corticosteroids provides a temporary benefit but can lead to important side effects.^8^^,^^9^ Currently we have surgery and topical steroids as the standard treatment, and biological as an option for failure cases.^10^ Dupilumab (Dupixent®, Regeneron Pharmaceuticals, NY, USA) is a humanized monoclonal antibody indicated for the treatment of various inflammatory diseases, such as atopic dermatitis, asthma, eosinophilic esophagitis, Chronic Obstructive Pulmonary Disease (COPD), prurigo nodularis, and CRSwNP. Its mechanism involves blocking the activity of the Interleukin-4 Receptor (IL-4Rα), modulating Interleukin-4 (IL-4) and Interleukin-13 (IL-13) signaling pathways, two key mediators of type-2 inflammation. Approved in Brazil in 2020 as an add-on maintenance treatment for adults with CRSwNP,^10^ dupilumab has demonstrated significant efficacy in phase III randomized, double-blind controlled trials (SINUS-24 and SINUS-52).^11^ These studies demonstrated that dupilumab improved both objective outcomes and Patient-Reported Outcomes Measures (PROMs), while it was generally well tolerated and safe. A subsequent analysis revealed that dupilumab significantly improved the sense of smell compared to placebo.^12^ Similar findings have been observed in real-world studies, reinforcing dupilumab’s effectiveness in clinical practice.^13^

Romano et al. identified a mixed inflammatory pattern in the Brazilian population, including both eosinophilic and neutrophilic responses, and classified it as high and low inflammatory patterns based on cytokine levels and eosinophil counts in tissue biopsies (cut-off: 43/high-power field) of nasal polyps. There was a correlation between severity of the disease and the level of tissue inflammation.^14^

While numerous studies have investigated the effects of monoclonal antibodies on olfactory function in patients with nasal polyps, few have explored the long-term efficacy (e.g., one-year outcomes), included data from multiple centers, especially in the Latin American population.

The aim of the present study was to present the clinical experience and outcomes of patients with difficult-to-control CRSwNP treated with dupilumab at referral centers across different regions in Brazil. The primary objective was to evaluate changes in olfaction, nasal polyp score, and in quality of life, thereby contributing to a broader understanding of the efficacy of dupilumab in Latin American patients.

## Methods

This study was approved by the Research Ethics Committee of the Clinical Hospital of Ribeirão Preto Medical School, University of São Paulo (USP), Brazil, under registration nº 34195120.7.0000.5440. The selected patients received treatment at different centers (Clinical Hospital of Ribeirão Preto Medical School, University of São Paulo; Clinical Hospital of Marilia Medical School; Clinical Hospital of Medical Sciences at the State University of Campinas; Clinical Hospital of Medical School of Federal University of Bahia; Clinical Hospital of Medical Sciences-State University of Rio de Janeiro; Clinical Hospital of Medical School of University of Brasilia; Clinical Hospital of Paulista Medical School - Federal University of São Paulo). A total of seven centers participated in this multicenter study. The number of patients enrolled per center was as follows: 18, 10, 9, 6, 4, 4, and 2, respectively. All participating institutions are public tertiary-level university hospitals in Brazil, ensuring a degree of homogeneity in the healthcare setting and clinical protocols. Each center enrolled patients who met the predefined inclusion criteria, based on national guidelines.

All enrolled patients were 18-years or older, had difficult-to-control CRSwNP, and presented documented evidence of type-2 inflammation in accordance with the Brazilian guidelines for the use of immunobiologicals in CRSwNP.^15^ CRSwNP was defined according to the 2020 European Consensus on Rhinosinusitis and Nasal Polyps.^1^ In addition, anterior rhinoscopy or nasal endoscopy confirmed the presence of polypoid lesions, which were biopsied and had anatomopathological findings consistent with inflammatory nasal polyps.

The clinical parameters suggestive of type-2 inflammation were symptom onset between the ages of 30–50, significant olfactory improvement with the use of oral corticosteroids, adult-onset asthma, intolerance to Nonsteroidal Anti-Inflammatory Drugs (NSAIDs), and the presence of nasal polyps.^15^ The laboratory parameters used to document type-2 inflammation included tissue eosinophilia of ≥10 cells per high-power field, blood eosinophilia of ≥150 cells per microliter, and total serum Immunoglobulin-E (IgE) levels of ≥100 IU/mL, as per established criteria. Patients who met the diagnostic criteria for CRSwNP and presented at least three clinical criteria and at least one laboratory biomarker indicative of type-2 inflammation,^15^ were included in the study.

In addition to the aforementioned criteria, immunobiological therapy was indicated for patients who met severity and inadequate disease control criteria despite appropriate medical and surgical treatment, according to the Brazilian guidelines for immunobiological use in CRSwNP.^15^^,^^16^

The severity parameters were assessed based on the presence of at least three of the following clinical criteria: (1) Moderate-to-severe nasal congestion, measured using a visual analog scale with a score of ≥ 5-points; (2) Severe hyposmia or anosmia, assessed with a validated olfactory test for use in Brazil; (3) SNOT-22 score >35-points; (4) Uncontrolled asthma; (5) Two or more courses of oral corticosteroids within one year; and (6) At least one prior endoscopic sinus surgery (except for patients with a formal surgical contraindication).

Only patients with documented disease extension (nasal endoscopy ‒ defined by Nasal Polyp Score (NPS) ‒ and computed tomography) were selected, for it could be possible to compare the evolution with treatment.

According to the criteria for the indication of biologic therapy established by the Brazilian guidelines, there is no specific threshold defined for NPS or CT scores. Nevertheless, more extensive disease ‒ characterized by higher NPS value or elevated Lund-Mackay score ‒ is typically assigned greater weight and is more likely to warrant a stronger indication for biologic treatment. In this context, no cut-off value for the NPS or Lund-Mackay CT score was established as an eligibility criterion in the present study.

Patients with the following conditions were excluded from the study: (1) Fungal rhinosinusitis; (2) Antrochoanal polyps; (3) Suspected or confirmed cystic fibrosis; (4) Suspected or confirmed primary ciliary dyskinesia; (5) Granulomatous diseases; (6) Benign or malignant tumors; (7) Unilateral disease; and (8) Incomplete medical records.

For this cohort only patients indicated to dupilumab were included, and they were assessed before treatment (T0) and one year after the medication was initiated (T1). The parameters evaluated in both times were: quality of life (SNOT-22), endoscopy score (NPS), and smell test (CCCRC). Adequate response in each parameter was considered as follows: (1) Improvement in SNOT-22: ≥9-points; (2) Improvement in olfaction (CCCRC): ≥1 degree; and (3) Reduction in Nasal Polyps (NPS): ≥2 points. Patients were considered good responders if they fulfilled the three criteria; mild-moderate responders if they presented one or two of the criteria above; and non-responders if they had no response. This classification was based on the response criteria outlined in the Brazilian guidelines for the use of immunobiologicals.^15^ Patients were classified as controlled, partially controlled, or uncontrolled based on assessments of nasal polyp extent, quality of life, and sense of smell at the T1 time point (Table 1).

**Table 1:** Criteria for defining disease control.

	Controlled (both NPS and SNOT-22 should be present)	Partially controlled (at least two criteria should be present)	Uncontrolled (if one of these criteria was present)
NPS	0‒2	3 or 4	≥ 5
SNOT-22	< 20	21‒40	> 40
CCCRC	Moderate hyposmia, mild hyposmia, or normosmia	Moderate hyposmia, mild hyposmia, or normosmia	Anosmia or severe hyposmia

The table outlines the criteria used to classify patients as controlled, partially controlled, or uncontrolled based on their NPS, SNOT-22 score, and smell test (CCCRC) results. NPS, Nasal Polyp Score; SNOT-22, Sinonasal Outcome Test; CCCRC, Connecticut Olfactory test.

All patients receiving dupilumab were concomitantly treated with nasal saline irrigation associated with either budesonide or another topical intranasal corticosteroid. For those with comorbid asthma, inhaled corticosteroids were continued ‒ predominantly in the form of budesonide/formoterol combination therapy. Short-acting beta-agonists (e.g., salbutamol) were reserved for acute asthma symptoms only. The use of antihistamines (such as loratadine, bilastine, desloratadine, or fexofenadine) was prescribed only when clinically indicated.

### Statistical analysis

Nasal polyp score, olfactory test (CCCRC), and SNOT-22 scores at baseline (T0) were compared with results at one year of treatment (T1). For the statistical analysis, categorical variables are presented as frequencies and percentages, while quantitative data are expressed as means with 95% Confidence Intervals or, alternatively, as medians with interquartile ranges, depending on the data distribution. The paired *t*-test and Wilcoxon signed-rank test were used to assess the changes between baseline and the one-year follow-up. In addition, the Mann–Whitney test was applied to evaluate the differences between AERD and non-AERD patients. All analyses were performed using *R* software (*R* Core Team, version 4.3.1), with a significance level of 5%. To compare symptom control at the beginning and end of the study, Bowker's Test was used (p < 0.0001).

## Results

This study evaluated the response of patients with severe CRSwNP who were treated with dupilumab for 12-months. The initial sample comprised of 56 patients. Three patients discontinued the use of dupilumab due to adverse effects, which were: severe headache, hypereosinophilia and erythema nodosum, as followed described.

Hypereosinophilia: one patient, aged 63, had pre-existing asymptomatic eosinophilia (1,500 cells/μL) before starting treatment. After three months of dupilumab, eosinophil levels increased to 3,000 cells/μL. Although the patient remained asymptomatic and other hypereosinophilic syndromes were ruled out, the clinical team opted to discontinue treatment as a precaution. Erythema nodosum: one patient, aged 55, developed erythema nodosum confirmed by skin biopsy after four months of treatment, leading to discontinuation. Persistent severe headache: one patient experienced intense headaches after each dupilumab injection, which were refractory to analgesics. Due to the persistence of symptoms, the patient chose to stop treatment. These dropouts accounted for 5.3% of the total cohort and were carefully monitored. While they reflect potential adverse effects of dupilumab, the overall dropout rate is low and unlikely to have significantly affected the study’s outcomes. Nonetheless, these cases highlight the importance of individualized risk-benefit assessment when initiating biologic therapy.

Thus, the final sample was 53 patients, 30 of whom were female (56.6%). The mean age was 52.0-years (Standard Deviation [SD = 12.6]; range: 19 to 76-years). Among them, 51 patients (96.2%) had asthma, and 33 (62.3%) had AERD.

**Fig. 1:**
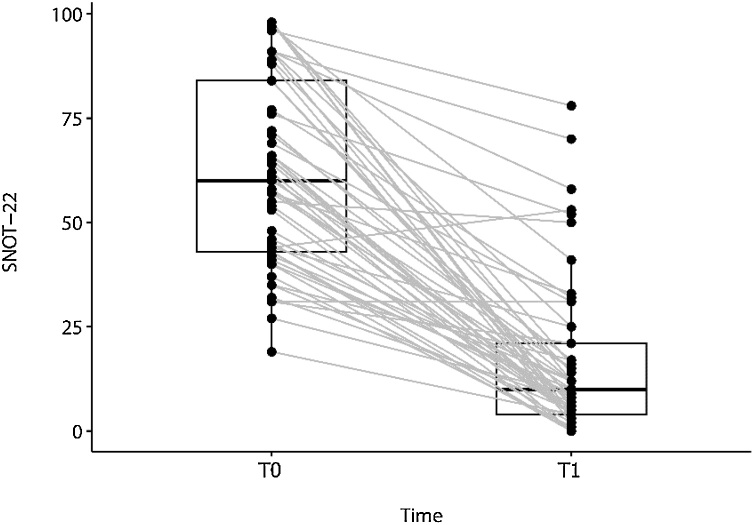
Changes from baseline (T0) SNOT-22 61.9 (95% CI 55.8‒68.1) to 16.7 (95% CI 11.5‒21.8) to one year following treatment (T1). Mean difference: 45.3 (95% CI 38.5‒52.1**)** (paired Student’s t-test, *t* = 13.4, p < 0.0001).

A significant reduction in SNOT-22 scores was observed, with the mean score decreasing from 61.9 (95% CI 55.8–68.1) to 16.7 (95% CI 11.5–21.8) at the end of the 1-year follow-up period. Mean difference: 45.3 (95% CI 38.5–52.1) (paired Student’s *t*-test, *t* = 13.4, p < 0.0001) (Fig. 1).

**Fig. 2:**
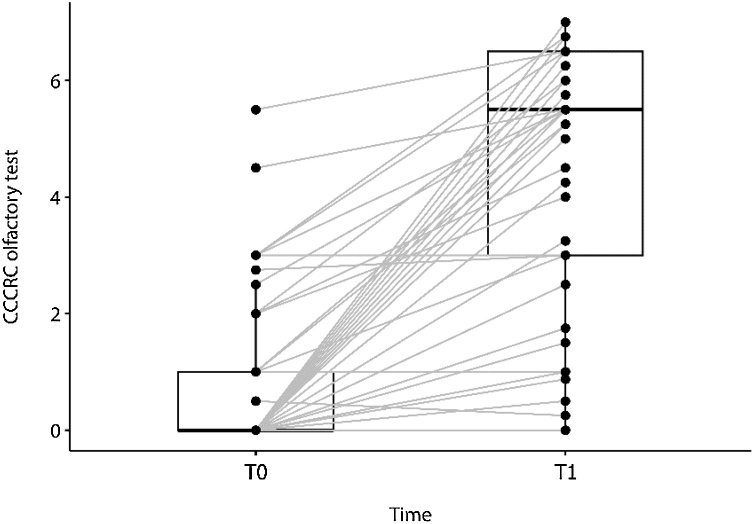
Changes from baseline (T0) in the CCCRC olfactory test (median: 0.0; P25‒P75 0.0‒1.0) to one year after treatment (T1); (median: 5.5; P25‒P75: 3.0‒6.5). Median difference: 4.25 (P25‒P75: 1.5‒6.0); Wilcoxon’s signed-rank test, p < 0.0001 (n = 53).

The CCCRC olfactory test showed a statistically significant improvement in the median score over the follow-up period, improving from anosmia (0-points) (median: 0.0; P25‒P75 0.0–1.0) to mild hyposmia (5.5-points) at the 1-year treatment (median: 5.5; P25‒P75: 3.0–6.5). Median difference: 4.25 (P25‒P75: 1.5–6.0) (Wilcoxon’s signed-rank test, p < 0.0001) (Fig. 2).

Among the 53 patients, 41 (77.3%) had anosmia at baseline (T0). At one year treatment with dupilumab (T1), 10 patients (24.4%) remained anosmic, while 31 (75.6%) improved by at least one severity level. Severe hyposmia was present in 9 patients (17%) at T0; of these, 2 (22.2%) remained unchanged, while 7 (77.7%) improved by at least one intensity level. Two patients (3.77%) had moderate hyposmia at T0, and both (100%) improved by one level at T1. Mild hyposmia was present in only one patient (1.88%) at baseline, who progressed to normosmia by the end of the study (Table 2).

**Table 2:** Distribution of patients by olfactory test category, at baseline (T0) and at 1-year follow-up (T1).

	At 1-year follow up (T1)					
Baseline (T0)	Anosmia, n (%)	Severe Hyposmia, n (%)	Moderate Hyposmia, n (%)	Mild Hyposmia, n (%)	Normosmia, n (%)	Total
Anosmia	10 (24.4%)	3 (7.3%)	3 (7.3%)	9 (22.0%)	16 (39.0%)	41
Severe Hyposmia	0 (0.00)	2 (22.2%)	2 (22.2%)	2 (22.2%)	3 (33.3%)	9
Moderate Hyposmia	0 (0.0%)	0 (0.0%)	0 (0.0%)	2 (100.0%)	0 (0.0%)	2
Mild Hyposmia	0 (0.0%)	0 (0.0%)	0 (0.0%)	0 (0.0%)	1 (100.0%)	1
Total	10	5	5	13	20	53

**Fig. 3:**
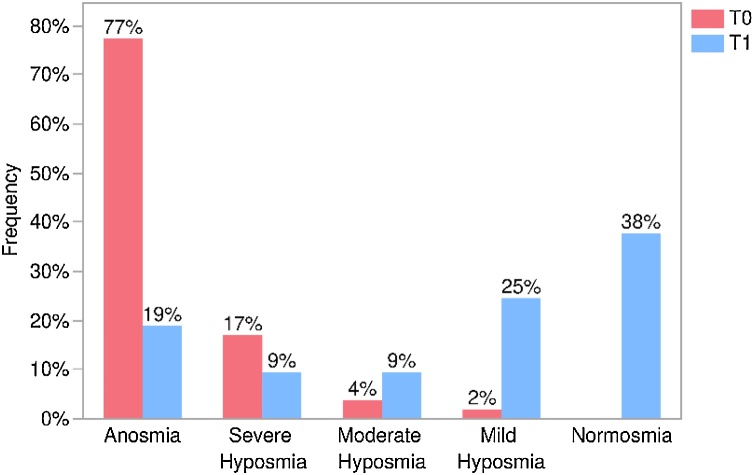
Changes in the distribution of patients across different olfactory test categories between the baseline period (T0) and one-year follow-up (T1).

Overall, at the 1-year follow-up, 10 patients (18.8%) had anosmia, 5 (9.4%) had severe hyposmia, 5 (9.4%) had moderate hyposmia, 13 (24.5%) had mild hyposmia, and 20 (37.7%) achieved normosmia (Fig. 2, Fig. 3).

**Fig. 4:**
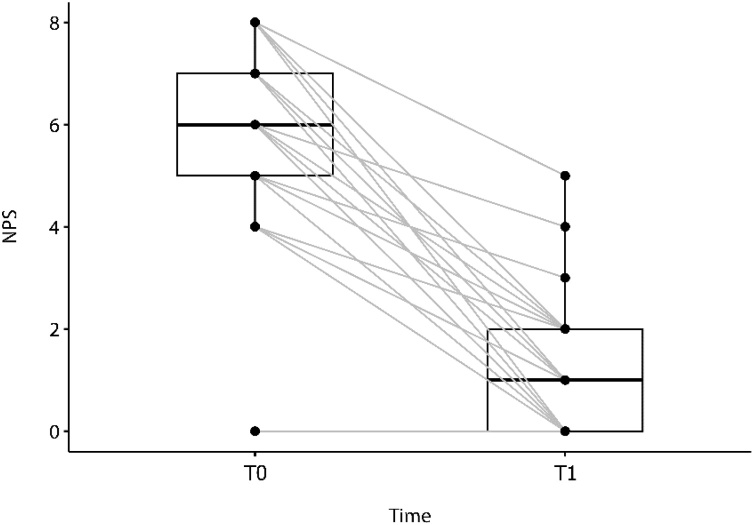
Changes from baseline (T0) Nasal Polyp Score (NPS) (median: 6.0; P25‒P75 5.0‒7.0) to one year (T1) following treatment (median: 1.0; P25‒P75: 0.0‒2.0). Median difference: 5.0 (P25‒P75: 4.0‒6.0); Wilcoxon’s signed-rank test, p < 0.0001 (n = 53).

Regarding NPS, we observed a decrease in the median score from T0: 6 (P25‒P75 5.0–7.0) to T1: 1 (P25-P75 0.0–2.0) after one year of treatment. The median difference was: 5.0 (P25‒P75 4.0–6.0). Interestingly, at the time of patients’ selection to medication, two patients had a NPS of 0, as they had recently undergone endoscopic nasal surgery. However, the immunobiologic treatment was indicated due to severe asthma and a significant impact on quality of life. A statistically significant difference was observed between baseline and the 1-year follow-up (Wilcoxon’s signed-rank test, p < 0.0001) for the NPS variable (Fig. 4).

Regarding the patients' responses to the use of dupilumab, all of them 53 (100%) presented favorable outcomes, with good response in 38 patients (71.7%) and mild-moderate response in 15 patients (28.3%). From those who had mild-moderate response, 13 patients fulfilled 2 and 2 patients fulfilled 1 of the established criteria.

**Fig. 5:**
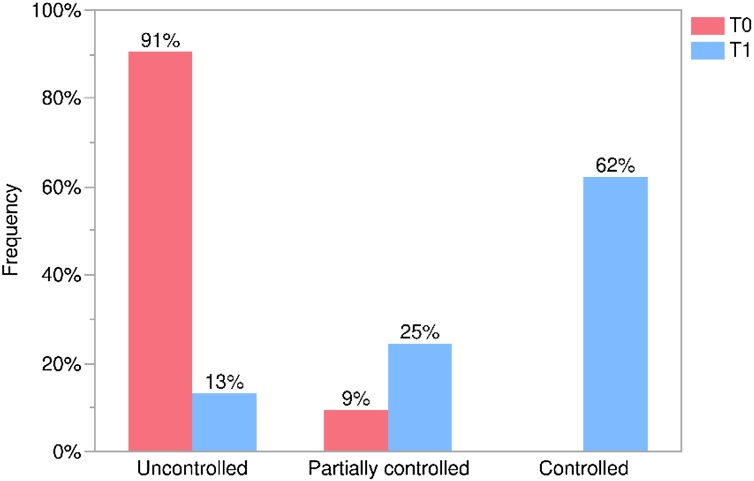
Changes in the distribution of patients regarding disease control in baseline period (T0) and one-year follow-up (T1).

In the analysis of CRSwNP control after treatment, only 7 patients (13.2%) remained in the “uncontrolled” condition, while the others showed some improvement, being classified as “partially controlled” 13 (24.5%) or “controlled” 33 (62.3%). Overall, 86.8% of the patients showed improvement (Fig. 5). A significant change in patient status was observed between the start of treatment and after 1-year (Bowker's Test, p < 0.0001).

Patients with AERD presented a similar response to dupilumab than those with only asthma (Table 3).

**Table 3:** Comparison of outcomes in AERD and non-AERD patients.

Difference	AERD (n = 20)			non-AERD (n = 33)			p-value^a^
	Median	IQR		Median	IQR		
		P25	P75		P25	P75	
Delta SNOT-22	47.5	30.0	63.0	41.0	24.0	58.0	0.35
Delta CCCRC	4.9	3.3	6.5	3.3	1.0	5.8	0.08
Delta NPS	5.0	4.0	6.3	5.0	4.0	6.0	0.85

Delta: difference between the values observed from baseline (T0) to 1-year after treatment (T1) with Dupilumab.

a Mann–Whitney test.

## Discussion

This study was the first to investigate the use of dupilumab in patients with CRS with nasal polyps (CRSwNP) in Latin America. Demonstrating the impact of dupilumab responses in these patients is crucial, especially in the current Brazilian context, in which the use of immunobiologics is growing, and with the prospect of some of these treatments being introduced into the public health system. This research was essential in assessing whether the outcomes observed in the Brazilian population align with those reported in real-world studies from other regions, particularly due to the characteristic mixed pattern of inflammation seen in the Brazilian population as described by Romano et al.^14^

In the phase III SINUS-24 and SINUS-52 studies,^11^ treatment with dupilumab in patients with severe CRSwNP led to significant improvements at weeks 24 and 52 compared to placebo across all evaluated outcome measures, including nasal polyp score, nasal congestion scale, SNOT-22 score and olfactory test. A recently published post-hoc analysis^17^ concluded that most patients with severe CRSwNP randomized in the SINUS studies had anosmia. Dupilumab treatment significantly improved olfactory outcomes compared to placebo, with 13.4% of the anosmic patients achieving normosmia. These prior results highlight the positive impact of dupilumab in addressing one of the most challenging and difficult-to-treat symptoms of CRSwNP.^17^

Real-world studies have also shown promising results with the use of biologics in patients with refractory type-2 CRSwNP.^18^, ^19^, ^20^ In a real-world European cohort study (CHRINOSOR)^18^ evaluating the efficacy of dupilumab in CRSwNP patients, improvements were observed in all outcomes from 24- to 52-weeks of treatment compared to baseline. Dupilumab demonstrated efficacy regardless of age, sex, body mass index, smoking status, number of prior nasal surgeries, presence of asthma, and AERD. A total of 92.5% and 94.4% of the individuals showed improvement in at least one EUFOREA criterion at 24 and 52 weeks, respectively, while 54.4% and 68.2% met the four most rigorous EUFOREA criteria at these time points. The real-world assessment of dupilumab’s efficacy demonstrated a robust and sustained response in at least two-thirds of the patients at 52-weeks.^18^

The multicenter, observational, real-world phase IV study DUPIREAL^19^ evaluated the efficacy and safety of dupilumab in patients with uncontrolled severe CRSwNP during the first year of treatment. The authors reported a significant reduction in nasal polyp scores, from a median value of 6 (IQR: 5–6) at baseline to 1 (IQR: 0.0–2.0) at twelve months (p < 0.001), and a significant reduction in SNOT-22 scores, from a median of 58 (IQR: 47–70) at baseline to 11 (IQR: 6–21) at twelve months (p < 0.001). The study results were not influenced by comorbidities, the number of prior surgeries, or adherence to topical corticosteroids. An excellent-to-moderate response was observed in 96.9% of the patients at twelve months based on EPOS 2020 criteria.^1^

A preliminary study of a prospective, observational, real-world cohort^20^ reported significant improvements in mean scores for all primary outcomes compared to baseline. The patients were evaluated at 24- and 48-weeks. SNOT-22 scores decreased from 52.4 (SD = 19.6) to 18.5 (12.9) and 16.8 (12.4), respectively. The nasal polyp score improved from 5.4 (2.0) to 1.6 (1.7) and 1.0 (1.7), while the olfactory test scores (Sniffin’ Sticks 12: 0–6 anosmia, 7–10 hyposmia, 11–12 normosmia) improved from 3.6 (2.1) to 7.3 (2.8) and 8.3 (3.2). At baseline, CRS was uncontrolled in 95.8% of patients, partially controlled in 4.2%, and fully controlled in 0%. At 24 and 48 weeks, respectively, 75.7% and 93.8% of patients were partially controlled, while 24.3% and 6.2% remained uncontrolled.

After 12-months of treatment with dupilumab, the patients in our cohort showed significant improvements in all the parameters evaluated. Olfactory function, quality of life, and the size and extent of nasal polyps improved markedly for the vast majority of patients. These improvements are consistent with findings reported in the literature.^12^^,^^13^^,^^17^, ^18^, ^19^, ^20^

Using the classification system for the general CRS condition based on the variables in this study, there was a significant positive change between the pre-treatment and 1-year treatment periods. Between T0 and T1, uncontrolled patients decreased from 48 (90.6%) to 7 (13.2%), partially controlled patients increased from 5 (9.4%) to 13 (24.5%), and controlled patients increased from 0 (0.0%) to 33 (62.3%). Although, in terms of treatment benefit, 53 patients showed some degree of response, with 38 (71.7%) showing complete response and 15 (28.3%) showing partial response. Based on the findings, our data supports the dupilumab’s benefits for CRS patients as previously reported in other real-world studies and controlled clinical trials.

During the data review process, we identified two patients who had recently undergone endoscopic sinus surgery but remained symptomatic and met the criteria for initiation of biologic therapy, particularly due to severe asthma and persistent olfactory dysfunction. The inclusion of these patients may have reduced the observed effect size regarding nasal polyp burden. Nevertheless, despite their inclusion, dupilumab remained effective in reducing nasal polyps overall and, notably, led to substantial improvement in olfaction in these specific cases.

Real-world studies, such as the present one, are essential for contextualizing patient responses to dupilumab in the treatment of chronic rhinosinusitis with nasal polyps. These findings provide valuable insights that can aid clinicians in making more informed therapeutic decisions and assist expert panels in refining the indications for specific biologic therapies on an individualized basis. This is particularly relevant when real-world evidence is compared across the spectrum of biologics currently available. Moreover, beyond guiding expert consensus, such data contribute meaningfully to the development of comprehensive databases that support clinical decision-making tools ‒ including those powered by artificial intelligence, such as ChatGPT ‒ as recently proposed and explored by Sireci et al., 2024.^21^

It is important to note that, as a multicenter study involving services with distinct follow-up protocols, this work is limited by the lack of additional data on disease control across the various patients, such as asthma control and the need for rescue oral corticosteroids during the treatment period. Also, the study design is observational and retrospective, limiting the ability to establish causal relationships. While patient selection in our study followed the criteria established by the Brazilian guidelines for the use of biologics in CRSwNP, it is important to note that these criteria are largely aligned and somehow overlapped with those of other international guidelines, such as EPOS/EUFOREA 2023.^22^ All of these recommend biologic therapy for patients with severe disease who remain symptomatic despite optimized medical and/or surgical treatment, and who exhibit clinical markers of type 2 inflammation. Therefore, we believe that our inclusion criteria are consistent with global standards and do not significantly limit the generalizability of our findings to other populations. Our data may, in fact, complement international studies by providing real-world evidence from an underrepresented region. Over one year of patient follow-up, all 53 individuals who continued treatment with dupilumab adhered to the recommended biweekly dosing regimen, demonstrating excellent adherence. However, data on prior or concomitant therapies were not available, representing one of the study's limitations.

In the future, continuous follow-up of these patients should be prioritized. Data enabling the evaluation of control, remission, and potential cure with dupilumab use will be monitored. This will not only help demonstrate the safety and efficacy of the treatment but also, in the long term, facilitate studies assessing dose spacing and the maintenance of patient responses.

## Conclusion

Our study shows that dupilumab effectively controls symptoms, improves quality of life and the sense of smell, and reduces nasal polyps’ burden after one year of treatment in Brazilian patients with hard-to-treat CRSwNP.

## ORCID ID

Otávio Marana Mieli: 0009-0003-3989-2886.

Fabiana Cardoso Pereira Valera: 0000-0002-6605-5317.

Vanessa Ramos Pires Dinarte: 0000-0001-7018-2397.

Clara Mônica Figueiredo de Lima: 0000-0001-5038-7977.

Marcio Nakanishi: 0000-0002-8010-3688.

Maria Eduarda Trocoli Zanetti: 0000-0002-6665-319X.

Felipe Oliveira Pires: 0009-0009-5631-2138.

Denny Marcos Garcia: 0000-0001-9123-2728.

Adriana de Andrade Batista Murashima: 0000-0002-8682-8495.

Luisa Karla de Paula Arruda: 0000-0002-7505-210X.

Fabrizio Ricci Romano: 0000-0002-2218-8417.

José Eduardo Seneda Lemos: 0000-0002-8714-129X.

Eduardo Macoto Kosugi: 0000-0003-1318-0541.

Eulalia Sakano: 0000-0002-5963-912X.

Miguel Soares Tepedino: 0000-0001-9000-9743.

Edwin Tamashiro: 0000-0002-3153-6292.

Wilma Terezinha Anselmo-Lima: 0000-0001-9146-2320.

## Funding

No funder.

## Declaration of competing interest

The authors declare that there is no conflict of interest regarding the publication of this paper. All research was conducted without any commercial or financial relationships that could be construed as a potential conflict of interest.
